# Editorial: Determinants of achievement in top sport

**DOI:** 10.3389/fpsyg.2026.1793950

**Published:** 2026-02-17

**Authors:** Donatella Di Corrado, Matej Tusak

**Affiliations:** 1 Department of Sport Sciences, Kore University, Enna, Italy; 2 Department of Social and Humanistic Sciences in Sport, University of Ljubljana, Ljubljana, Slovenia

**Keywords:** elite sport, leadership, motivation, performance, personality, sport psychology

Achieving excellence in top-level sport represents the outcome of a complex interaction among physical, technical, psychological, motivational, and contextual factors. While athletic success has traditionally been explained through physical preparation and technical mastery, contemporary research increasingly highlights the decisive role of psychological determinants in sustaining elite performance and facilitating long-term achievement. In line with the social-cognitive perspective that currently dominates psychological research, motivation, self-regulation, emotional control, and environmental influences have become central constructs in the study of elite sport.

Numerous studies have probed the key factors behind success. The rise of the current trend in social cognitive psychology has pivoted attention toward motivation in high-level athletics. Social cognitive theories now dominate research across nearly all areas of psychology.

Although motivation and personality have been widely studied and debated, solid evidence linking psychological training, pre-competition mental states, and specific mental strategies to athletic performance and success remains limited. Crucial uncertainties remain in pinpointing which psychological methods and techniques drive excellence in top-tier sports. Upcoming regression analyses aim to dissect success drivers by contrasting male and female athletes, as well as youth competitors against elite performers. The precise equation for victory—balancing pre-competitive mindset, mental conditioning, technical prowess, and expertise—stays elusive. Methods such as hypnosis, mental imagery, and cognitive strategies for stress management remain underexplored. Bridging this research void is essential.

Within this framework, the Research Topic Determinants of Achievement in Top Sport was designed to collect and integrate scientific contributions addressing the psychological, motivational, and organizational factors associated with success, persistence, and excellence in high-performance sport. The articles included in this collection examine these determinants across different sports, competitive levels, and cultural contexts, offering a multidimensional perspective on elite achievement.

The contributions can be broadly grouped into five main thematic areas, which are outlined below.

## Psychological and motivational determinants

Motivation and self-belief emerge as fundamental components of achievement in top sport. Several studies in this Research Topic emphasize the role of self-efficacy as a central psychological mechanism underlying performance, engagement, and persistence. Zhang and Khan examined Chinese university athletes, showing that self-efficacy is positively associated with performance through the mediating roles of psychological resilience and emotional regulation, with autonomy-supportive coaching acting as a significant moderator. Similarly, Chen et al. demonstrated that psychological resilience mediates the relationship between parenting styles and athletic performance in adolescent athletes, highlighting the importance of motivational climates across developmental stages. Feng et al.' chain mediation model revealed that social support helps Chinese university-level soccer players manage career anxiety effectively. This relationship is sequentially mediated by career choice self-efficacy and career planning.

The relationship between motivation, social support, and self-perception is further explored by Huang and Li, who found that athletes' self-esteem and perceived social support are linked through adaptive cognitive mechanisms such as decentering, while Wei et al. showed that emotion regulation, self-efficacy, and social support contribute differently to resilience in team vs. individual sports during adolescence.

Intrinsic motivation and optimal psychological functioning are also addressed in studies focusing on flow and sensation seeking. Mancin et al. investigated Italian motorcycle racers, revealing that intrinsic motivation and dispositional flow are key psychological drivers in high-risk sport contexts. Complementarily, Lee and Hwang highlighted the psychological strategies underlying the success of elite South Korean archers, emphasizing the role of motivation, focus, and mental discipline in precision sports.

## Stress, emotional states, and performance

Managing stress and emotional demands is a defining challenge in elite sport. Several contributions in this Research Topic address how athletes perceive and regulate competitive stress and how emotional states influence performance outcomes. Pepe et al. demonstrated that positive thinking skills shape athletes' perceptions of excellent performance through challenge and threat appraisals. Complementing this cognitive perspective, Di Corrado et al. further examined the relationship between mental imagery, stress, and self-efficacy in martial arts athletes, showing that imagery contributes to performance by strengthening athletes' confidence and stress management abilities. Extending these findings, Robazza et al. examined dispositional mindfulness within the multi-states framework, showing that mindful awareness and refocusing foster challenge appraisals and pleasant, functional psychobiosocial experiences while reducing negative emotional states. Together, these studies highlight a shared mechanism whereby cognitive and attentional strategies—such as positive thinking, imagery, and mindfulness—facilitate adaptive appraisals and emotional regulation, ultimately supporting performance under competitive stress.

Extending the focus on psychological processes beyond athletes to the broader sport context, Yang et al. investigated how the image attributes of golf star athletes influence adult amateur golfers' desire to participate in the sport and their intention to continue. Their findings suggest that positive emotional and cognitive responses—elicited by athletes' image attributes—also play a crucial role in shaping motivation and sustained engagement in sport, underscoring the shared psychological mechanisms linking stress regulation, emotional appraisal, and sport participation.

Pre-competitive psychological states are also examined across different competitive levels and genders. Kelemen et al. investigated mental preparation in runners, identifying gender differences and competition-level effects on psychological training and performance outcomes. Similarly, Wang, Li et al. conducted a gender-stratified analysis of badminton athletes, identifying psychological predictors that distinguish competitive levels, thus reinforcing the relevance of emotional and cognitive readiness before competition.

Sports performance analysis has become a cornerstone of modern athletics. It blends data-driven insights with expert observation to optimize athlete training, refine techniques, and predict outcomes. Gan et al. tested subjective evaluation reliability for elite table tennis under varying info conditions and observer expertise effects. Skilled observers showed high personal reliability but low inter-observer agreement; consistency fell without kinematics/results, varied by indicators, and rose with experience/skill.

## Personality, burnout, and athlete development

Personality traits and long-term psychological functioning represent another core theme of this Research Topic. Fu et al. conducted a longitudinal study on self-oriented perfectionism and athlete burnout, demonstrating that maladaptive perfectionism is associated with increased burnout risk over time. Gómez-López et al. further explored perfectionism and fear of failure among handball referees, highlighting how sporting experience shapes personality-related stress responses.

Extending this focus on stable personality characteristics to risk-related behaviors, Guo et al. validated the Contextual Sensation-seeking Questionnaire for Skiing and Snowboarding among Chinese adult skiers and examined the link between sensation seeking and risk-taking behavior. Their findings confirm that the instrument is reliable and valid and show that higher sensation seeking is associated with greater injury frequency, with risk perception acting as a mediating factor. The issue of athlete development and retention is addressed in depth by Brusvik and Söderström, who investigated reasons for dropout among selected young female and male football players on their path to elite senior levels. Their findings underline the importance of psychological, organizational, and motivational factors during critical career transitions and stress the need for supportive developmental environments.

From a longitudinal perspective, early cognitive and physical foundations may play a crucial role in shaping long-term athletic trajectories. In this regard, Alghamdi et al. showed that physical fitness in childhood is positively associated with executive functions, particularly working memory, highlighting the importance of early physical activity for cognitive development.

Building on this developmental pathway into elite sport, Boone et al. demonstrated that cognitive abilities assessed later in athletes' careers—such as reaction time, visual–spatial processing, and decision making—significantly enhance the prediction of professional performance in NFL quarterbacks beyond draft position. Together, these findings suggest a continuum in which early cognitive–physical development may underpin the advanced cognitive skills required for elite performance at the professional level.

Achievement in sport refers to the attainment of performance goals and competitive success, resulting from the interaction of personal, psychological, and environmental factors that support athletes' development and performance. Zhu Y. et al. aimed to examine how achievement goal orientation (AGO) influences swimmers' perceived performance through multiple psychological mediators. Their findings show that AGO positively affects perceived performance mainly through a sequential pathway involving sports enthusiasm, sports commitment, and grit, highlighting the crucial role of these psychological factors in enhancing athletic performance.

Similarly, Zhu D. et al. investigated how family quality of life influences youth badminton athletes' achievement through athlete identity. The results indicate that family quality of life positively affects achievement both directly and indirectly by strengthening athlete identity, while also revealing that psychological and emotional family support tends to lag material support and therefore requires greater attention.

## Coaching, leadership, and organizational contexts

Beyond individual psychological characteristics, several contributions emphasize the decisive role of coaching behaviors, leaders, and sport organizations in shaping achievement. Kwon and Kim showed that instructors' service quality influences learning transfer through athletes' positive and negative emotions, emphasizing emotions as a key mechanism linking coaching actions to performance. Complementing this applied perspective, Kolar et al. provided a conceptual framework that clarifies how coaches' strategic, tactical, and operational decisions are grounded in distinct cognitive processes and leadership styles across training and competition contexts.

Building on these foundations, several empirical studies underline the importance of leadership and relational dynamics. Peng et al. demonstrated that transformational leadership enhances team cohesion via mental toughness, while Kim showed that authentic leadership fosters trust, self-efficacy, service quality, and long-term coach–athlete relationships. Similarly, Li and Xing found that coach leadership behavior and achievement goal orientation predict athlete engagement through the mediating role of basic psychological needs, highlighting leadership as a particularly malleable factor. Finally, Dai et al. extended this relational focus by showing that coach–athlete attachment shapes engagement through thriving, with mental toughness moderating these effects. Collectively, these findings suggest that effective coaching integrates decision-making, leadership style, emotional processes, and relational sensitivity to optimize athlete engagement and performance. Wang, Xu et al. examined how team cohesion influences athlete engagement in collegiate basketball and how different paternalistic leadership styles moderate this relationship. The findings show that team cohesion positively predicts athlete engagement, while authoritarian leadership weakens this effect and moral leadership strengthens it, highlighting the importance of ethical and supportive coaching styles in fostering engaged and cohesive teams.

The influence of leadership style on athlete engagement is further explored by Li et al., who identified basic psychological needs as key mediators between coaching leadership behaviors and athlete engagement. At the organizational level, Monton et al. provided a case study of Speed Skating Canada, illustrating how people-centered organizational practices can successfully coexist with high-performance objectives. Brinkmöller et al. sought to examine the links between talent selection research in sports and business. They found that the two fields are largely disconnected and have developed independently, despite similar goals. However, cross-contextual links have increased since 2018, indicating a growing interest in integration.

## Contemporary challenges and the COVID-19 era

Finally, this Research Topic addresses contemporary challenges affecting elite sport. Aase et al. explored the experiences of Norwegian Olympic handball players during the COVID-19 pandemic, highlighting how psychological resilience and adaptive coping strategies supported athletes' journey from disruption to elite performance. Complementarily, Xiong et al. provided a data-driven analysis of the effects of COVID-19 infection on the performance of top basketball players, offering valuable insights into the long-term implications of pandemic-related stressors. Alarfaj et al. aimed to explore the developmental pathways leading to performance excellence in twice-exceptional Paralympic athletes. The findings identify two key stages of development—an initial phase driven by spontaneous motivation and a later phase characterized by structured training and goal setting—highlighting the dynamic interaction of personal, psychological, social, and contextual factors in achieving elite performance.

## Conclusion

To conclude, the studies included in this Research Topic provide strong support for the multidimensional nature of success in top sport. By integrating psychological, motivational, developmental, leadership, and contextual perspectives, this collection advances current knowledge on elite performance and offers valuable implications for researchers, practitioners, coaches, and sport organizations. We hope that the findings, insights, and perspectives presented in this Research Topic will inspire future research and contribute to the continued development of evidence-based practices in elite sport.

